# NLRP12 in autoimmune diseases

**DOI:** 10.18632/oncotarget.4585

**Published:** 2015-06-22

**Authors:** Prajwal Gurung, Thirumala-Devi Kanneganti

**Affiliations:** Department of Immunology, St Jude Children's Research Hospital, Memphis, TN, USA

**Keywords:** immunology and microbiology section, immuneresponse, immunity

Nucleotide-binding oligomerization domain (NOD)-like receptors (NLRs) are a group of cytoplasmic sensors that survey danger signals released by invading pathogen or damaged self-tissues. NLRs can be categorized into subgroups based on their ability to form inflammasome, potentiate inflammatory signaling, act as transcriptional regulators and negatively regulate inflammatory pathways. NLRP12, first described about 10 years ago, is a cytoplasmic sensor to be categorized as a negative regulator of inflammation. A recent study has also shown that NLRP12 can form an inflammasome during infection with *Yersinia pestis*, although this study needs to be substantiated by independent groups [1].

Two seminal studies in 2011 showed that NLRP12 is a crucial negative regulator of NF-κB signaling pathway [2, 3]. The absence of NLRP12 in mice resulted in severe uncontrolled inflammation that rendered these NLRP12deficient mice highly susceptible to experimental colitis and inflammation-induced tumorigenesis [2, 3]. NLRP12 was crucial in both hematopoietic and non-hematopoietic compartment for controlling overt inflammation, colitis and colitis-associated tumorigenesis. We have further shown that NLRP12 also negatively regulates NF-κB signaling during *Salmonella* infection to limit inflammation [4]. While the etiology of infectious diseases, colitis and cancer require coordination of several immune and stromal cells, most of the mechanistic insights on the molecular underpinnings of NLRP12 have only been conducted in innate immune cells that include macrophages and dendritic cells. Specifically, T cells are central to the regulation of all of these disease manifestations but the role of NLRP12 in T cells was not known.

Analysis of NLRP12 expression in various cell populations revealed that T cells express relatively higher levels of NLRP12 in comparison to both dendritic cells and macrophages [5]. While NLRP12 was not required for negative and positive selection of T cells in the thymus, our studies showed that *Nlrp12*^−/−^T cells produce higher levels of Th1 (IFN-γ), Th2 (IL-4) and Th17 (IL17) cytokines following *ex vivo* stimulation, suggesting a cell intrinsic role for NLRP12 in negatively regulating T cell activation [5]. In agreement, in a T cell transfer-induced colitis model, mice receiving *Nlrp12*^−/−^ T cells exhibited dramatically severe colitis when compared to mice receiving WT T cells. In addition to severe colitis, mice receiving *Nlrp12*^−/−^T cells also displayed severe inflammation of the skin and ears, i.e. atopic dermatitis. Further analysis of cytokines in these diseased mice showed that the disease transferring *Nlrp12*^−/−^T cells also produced excessive levels of Th2 cytokines such as IL4, IL-5 and IL-13, which are critical in promoting atopic dermatitis. Surprisingly, *Nlrp12*^−/−^ mice showed protection from classical disease symptoms of experimental autoimmune encephalitis (EAE), a T cell driven autoimmune disease that mimics multiple sclerosis in humans. Closer observation revealed that *Nlrp12*^−/−^mice suffered from non-classical symptoms of EAE that include loss of balance and ataxia. Interestingly, the atypical EAE phenotype displayed by *Nlrp12*^−/−^mice are symptoms that are more prevalent in humans and could be attributed to excessive IL-4 production by T cells. Thus, *Nlrp12*^−/−^ mice present a much more realistic model to understand multiple sclerosis in humans. Anti-IL-4 neutralizing antibodies treatment reversed the loss of balance and ataxia symptoms of *Nlrp12*^−/−^mice. Mechanistically, we showed that NLRP12 operates downstream of TCR-signaling to negatively regulate the NF-κB signaling pathway and inhibit production of all major T cell cytokines [5]. The importance of our studies is highlighted by human genetic studies where missense mutations in the *NLRP12* gene have been reported to result in severe autoinflammatory diseases highlighted by periodic fever syndrome and atopic dermatitis [6, 7]. Our studies suggest that understanding and targeting T cells in these patients with NLRP12 mutations might be a potential therapeutic approach. Now that we have shown NLRP12 also plays an important role in EAE, screening human multiple sclerosis patients for mutations in the *NLRP12* gene will be important to understand this debilitating autoimmune disease.

Using two different T cell driven autoimmune disease models (T cell driven colitis and EAE), we showed that NLRP12 plays central roles in T cells to control overt inflammation and maintain cellular homeostasis [5]. The exact molecular mechanisms of how NLRP12 tame NF-κB activation in T cells will need to be studied. It should be noted that molecular pathways leading to the activation of macrophages/dendritic cells by TLR ligation is completely different from activation of T cells by TCR ligation; yet NLRP12 is able to negatively regulate NF-κB activation in both of these cell types. It will be interesting to examine whether NLRP12 employs similar molecular strategies to block NF-κB activation in T cells compared to macrophages/dendritic cells. While the use of adoptive transfer, passive transfer and chimeras were invaluable in demonstrating T cell intrinsic role for NLRP12 in our studies [5], generation of NLRP12-conditional knockout mice (*Nlrp12*^flox/flox^ mice) will be central in deciphering the precise role of NLRP12 in T cells. The role of NLRP12 in various T cell subsets (naïve, activated, memory, regulatory) is completely unknown and needs further investigation. Importance of NLRP12 in T cells during acute and chronic infections or vaccine treatments are all open questions that will need to be examined as well. In addition, the role of NLRP12 in other immune cells such as B cells, NK cells and gamma delta T cells are completely unknown and NLRP12 conditional mice will be instrumental in these studies. The roles of NLRP12 are only beginning to be uncovered and the complete understanding of how NLRP12 regulates inflammation in various disease settings, immune cells and inflammasomes will undoubtedly lead to novel treatment therapies.
